# Implementation of a protocol for predicting successful extubation in critically ill patients

**DOI:** 10.1186/2197-425X-3-S1-A316

**Published:** 2015-10-01

**Authors:** P Guijo González, Á Estella, JM Ramos Rodríguez, T Rico Armenteros, M Jaen Franco, M Recuerda, A Jareño Chaumel, L Fernández Ruiz

**Affiliations:** Hospital del SAS de Jerez, Intensive Care Unit, Jerez de la Frontera, Spain

## Introduction

Weaning protocols have been show to be better than traditional physician directed discontinuation of mechanical ventilation. Extubation failure can cause increased complications, higher ICU length of stay, higher cost, morbidity and mortality.

## Objectives

The aims of the present study are to analyze the influence of implementation an extubation protocol and to compare clinical outcome with a traditional strategy based in the physician´s criteria for discontinue mechanical ventilation.

## Methods

Observational study performed in a 17 beds medical-surgical ICU. Time of study was 18 months. Consecutive mechanically ventilated patients during more than 48 hours were included. The variables analyzed were age, sex, cause of the intubation, APACHE II at admission in ICU, vasoactive requirements, fluid balance, day of weaning, ICU length of stay, tracheotomy, rate of reintubation. We distinguish two subgroups according extubation strategy, based in physician´s criteria vs protocol designed by a intensivist and nursing team including haemodynamic and respiratory parameters like: negative inspiratory force, maximal inspiratory pressure, f/V_T_, airway occlusion pressure at 0,1 second. The data collected were analyzed using SPSS version 22 for Windows.

## Results

91 patients were analyzed, 54 were extubated based on the protocol and 37 according physician criteria. There were not differences in clinical characteristics, APACHE score, fluid balance and vasoactive requirements.

Reintubation rate was higher (35.1% vs 5.6%) in not protocolized group with a statistical significant difference (RR, 0.109; 95% CI 0.028-0.417). Tracheotomy requirements, ICU lenght of stay and mortality was lower in the protocol -directed ventilator weaning group.

## Conclusions

Physician directed extubation was associated with an increase in the rate of reintubations and ICU length of stay compared with a protocolized weaning strategy.

**Table 1:** Study characteristics

	Protocol (n: 54)	Physician criteria (n:37)
Age	63	58
APACHE II at admission	20	19
24 h fluid balance (accumulated)	-1155 ml(184)	-1502 ml (-336)
Ventilator Associated Pneumonia (%)	5.6%	10%
ARDS (%)	7.4%	16%
Tracheotomy (%)	3.7%	5.4%
ICU lenght of stay	10.17	14.14
Reintubation (%)	5.6%	35.1%
Mortality (%)	3.7%	5.4%

